# Kinematical error analysis and autonomous calibration of a 5PUS-RPUR parallel robot

**DOI:** 10.1371/journal.pone.0330675

**Published:** 2025-09-02

**Authors:** Zesheng Wang, Yanbiao Li, Bo Chen, Kexin Ding, Jialong Zhu, Min Zhuang

**Affiliations:** 1 School of Intelligent Manufacturing, Hangzhou Polytechnic, Hangzhou, China; 2 State Key Laboratory of Fluid Power and Mechatronic Systems, Zhejiang University, Hangzhou, China; 3 College of Mechanical Engineering, Zhejiang University of Technology, Hangzhou, China; Beijing Institute of Technology, CHINA

## Abstract

Kinematic calibration is essential for improving the absolute accuracy of parallel robots, but conventional identification methods often struggle with the complex, non-linear coupling of their numerous geometric error parameters. This can lead to convergence to local rather than global optima, limiting the effectiveness of the calibration. To address this challenge, this paper proposes a novel self-calibration methodology based on a global optimization strategy. Taking the 5PUS-RPUR parallel robot as an example, its inverse kinematics is established based on screw theory. A sensitivity analysis is performed using the finite difference method to screen for and eliminate error sources with a negligible impact on the moving platform’s pose. Measurement points are then selected uniformly throughout the workspace using the farthest point sampling algorithm. An objective function for the GA is constructed by integrating the actuator displacement errors from each kinematic chain with the overall pose error of the moving platform. Non-linear constraints are handled using a penalty function approach. Based on measurement data from an onboard IMU and joint encoders, the identification results are obtained. The experimental results demonstrate that the proposed method significantly improves the robot’s positional accuracy across its entire workspace. The superiority and efficacy of this approach are further corroborated by a benchmark comparison with three recent, state-of-the-art calibration methodologies.

## 1. Introduction

Lower-mobility parallel robots offer advantages such as simpler architecture and lower costs in design, control, and manufacturing compared to their 6-DOF counterparts [1]. They are well-suited for numerous tasks requiring fewer than six DOFs in industrial and medical applications [2]. For instance, 3T2R motion capabilities cover a wide range of applications, including 5-axis machining [3,4], welding [5], and surgical procedures [6]. However, physical robots are unavoidably subject to geometric imperfections, such as manufacturing tolerances and link misalignments, which cause significant discrepancies between the commanded and the actual end-effector pose. Calibration offers a powerful and cost-effective method to compensate for these deviations through software, thereby enhancing pose accuracy without requiring expensive improvements in manufacturing precision [7–9].

Kinematic calibration generally involves four key steps: error modeling, measurement, parameter identification, and compensation [10]. First, an error model is formulated to relate the robot’s nominal kinematics to its actual behavior, typically using data from internal or external sensors. Based on this model, a parameter identification process is then performed to quantify the geometric errors [11–13]. Subsequently, the identified parameters are used to modify the controller model, thereby improving the pose accuracy of the parallel robot [14,15]. However, the large number of error parameters and their complex, non-linear coupling in parallel robots significantly complicate the identification process. To address these challenges, numerous researchers have explored advanced identification strategies, motivating the work presented in this paper. Luo et al. [16] developed a kinematic model for a novel 4PPa-2PaR parallel manipulator that incorporates its non-ideal geometric parameters. They employed the Levenberg-Marquardt (LM) algorithm to identify 10 key error parameters, with the objective of minimizing the error in each individual kinematic chain. After calibration, the average pose accuracy of the moving platform was greatly improved. Song et al. [17] proposed a robust calibration method for joint compensation based on an artificial neural network (ANN). Taking a Stewart platform as their case study, they set the minimization of individual kinematic chain errors as the optimization objective. The calibration experiments demonstrated a 91.90% reduction in the mean position error and a 90.22% reduction in the mean posture error. Gao et al. [18] established a linear error model for individual kinematic chains with the objective of minimizing inverse kinematic residuals. By combining full-pose measurements with actuator displacement data, they employed an iterative linear least-squares method for parameter identification. This approach reduced the pose error from 8 mm/ 0.4° before calibration to 0.4 mm/ 0.04° after calibration. Using screw theory, Sun et al. [19] established a comprehensive error model that included 18 geometric error sources of a 3-DoF rotational parallel manipulator. They then solved the error equation for each individual chain by combining Tikhonov regularization with the Generalized Cross-Validation (GCV) method for optimal parameter identification. Post-calibration results showed an improvement in orientation accuracy of over 53.4%. Zhang et al. [20] constructed a non-linear error model for the individual chains of a Stewart platform using closed-loop vector constraints. They employed the LM algorithm to solve the resulting non-linear least-squares problem. The experimental results demonstrated a significant improvement in accuracy: the position error was reduced from the millimeter level to within ±0.2 mm, while the orientation accuracy improved by an order of magnitude compared to the uncalibrated system. He et al. [21] presented a calibration methodology for a 7-DOF UP&2UPS-4R hybrid manipulator. A comprehensive error model was established by treating the parallel mechanism as an equivalent serial chain. An iterative least-squares method was employed to identify the kinematic parameter errors. This approach reduced the average error of the hybrid manipulator by 85%, significantly enhancing its kinematic accuracy. Existing strategies for error parameter identification, however, possess certain limitations. While optimization algorithms such as gradient descent, the Newton-Raphson method, and least-squares are computationally efficient, they typically employ an independent chain optimization strategy. This approach can only guarantee a local optimum for each individual kinematic chain, as it neglects the mutual constraint relationships between them. Consequently, the overall calibration result may deviate significantly from the global optimum.

Due to the high-order non-linearities and strong coupling among the various chain error parameters, obtaining an explicit mathematical model of their interdependencies is exceedingly difficult. This limitation necessitates an optimization approach capable of navigating the complex solution space without relying on a predefined coupling model. To this end, we turn to the Genetic Algorithm (GA), a metaheuristic renowned for its powerful global search capabilities and its adeptness at handling non-linear, multi-modal problems. The GA has demonstrated significant success in diverse fields such as the optimal allocation of electric vehicle charging stations [22], mobile robot path planning [23], and aircraft mission planning [24]. To achieve a globally optimal calibration for the parallel robot, we design a weighted fitness function that fuses the actuator displacement errors from individual chains with the overall pose error of the robot’s end-effector. This function, serving as the GA’s optimization objective, simultaneously evaluates the contribution of all chains to the end-effector accuracy during the optimization process. It thereby compels the algorithm to find a parameter set that holistically coordinates error compensation across the entire robot, fundamentally circumventing the issue of explicit parameter coupling and achieving a truly global optimum.

This paper presents the error modeling and kinematic calibration of a five degree-of-freedom 5PUS-RPUR (R, P, S, and U stand for revolute, prismatic, spherical, and universal joints) parallel robot, with the implementation by the following steps: (1) formulation of the inverse kinematics by integrating screw theory, Paden–Kahan (PK) subproblems and the elimination method; (2) error modeling within the screw theory framework and global sensitivity analysis of the influence of the geometric variations onto the moving platform pose; (3) identification of geometric parameters by pose measurement using a IMU and encoder. With the objective function that integrates individual chain actuator errors with the end-effector pose error, the parameter identification is realized by solving an unconstrained nonlinear optimization problem via GA. After calibration, the position error of the moving platform is significantly improved throughout the operational workspace.

## 2. Analysis of inverse kinematics

The inverse kinematics problem involves determining the required actuator displacements to achieve a desired end-effector pose. While a common approach for this analysis is to combine screw theory with Paden-Kahan (PK) subproblems, this method is not directly applicable to the 5PUS-RPUR robot, specifically for the inverse kinematics of its RPUR chain. To address this limitation, the elimination method is used to reduce the unknown parameters in the pose analysis of the RPUR chain.

**Fig 1:**
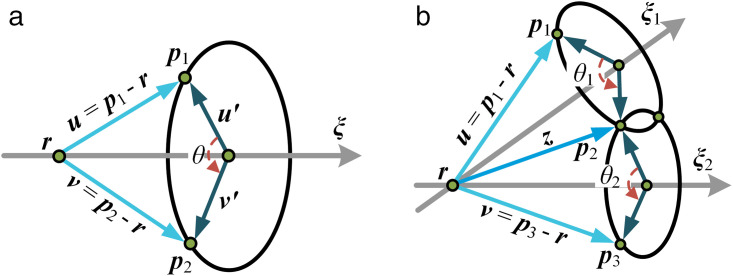
The two recognized basic PK sub-problems. **(a)** PK sub-problem 1. **(b)** PK sub-problem 2.


There are three recognized basic PK subproblems [25]. For the inverse position analysis of the 5PUS-RPUR parallel robot, the solution does not involve PK subproblems 3. This study only clarifies the first two recognized subproblems. PK subproblem 1 is shown in Fig 1(a), the spatial point ***p***_1_ rotates around the fixed axis ***ξ*** to the given point ***p***_2_, and ***r*** is a point on the axis ***ξ***. *θ* is the rotation angle to be calculated.

$$\begin{array}{c} \left\{ \begin{array}{c} \theta =\text{atan2}\left(\xi^{\text{T}}\left(u^{\prime }\times v^{\prime }\right),{u^{\prime }}^{\text{T}}v^{\prime }\right)\\ u^{\prime }=u-\xi \xi^{𝐓}u\\ v^{\prime }=v-\xi \xi^{𝐓}v \end{array}\right.\; \end{array}$$ (1)

PK subproblem 2 is shown in Fig 1(b), the spatial point ***p***_1_ rotates around axis ***ξ***_2_ and axis ***ξ***_1_ to the given point ***p***_3_, respectively, and *θ*_1_ and *θ*_2_ are the rotation angles to be calculated.


$$\left\{ \begin{array}{c} z=\iota_{\text{1}}\xi_{\text{1}}+\iota_{\text{2}}\xi_{\text{2}}+\iota_{\text{3}}\left(\xi_{\text{1}}\times \xi_{\text{2}}\right)\\ \iota_{\text{1}}=\frac{\left(\xi_{\text{1}}^{\text{T}}\xi_{\text{2}}\right)\xi_{\text{2}}^{\text{T}}u-\xi_{\text{1}}^{\text{T}}v}{{\left(\xi_{\text{1}}^{\text{T}}\xi_{\text{2}}\right)}^{\text{2}}-\text{1}},\iota_{\text{2}}=\frac{\left(\xi_{\text{1}}^{\text{T}}\xi_{\text{2}}\right)\xi_{\text{1}}^{\text{T}}v-\xi_{\text{2}}^{\text{T}}u}{{\left(\xi_{\text{1}}^{\text{T}}\xi_{2}\right)}^{\text{2}}-\text{1}}\\ \iota_{\text{3}}^{\text{2}}=\frac{{\left\|u\right\|}^{\text{2}}-\iota_{\text{1}}^{\text{2}}-\iota_{\text{2}}^{\text{2}}-\text{2}\iota_{\text{1}}\iota_{\text{2}}\xi_{\text{1}}^{\text{T}}\xi_{\text{2}}}{{\left\|\xi_{\text{1}}\times \xi_{\text{2}}\right\|}^{\text{2}}} \end{array}\right.\;$$ (2)

**Fig 2:**
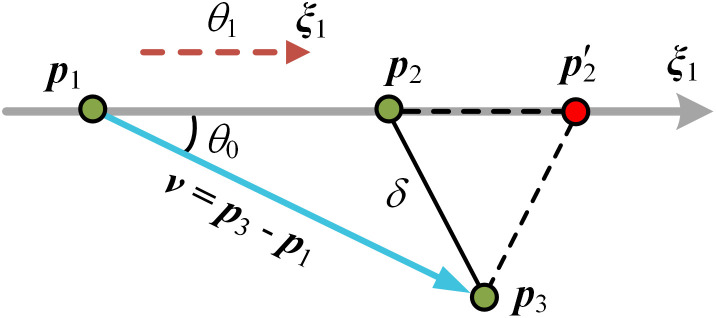
PK subproblem 3.


In addition to the known P-K sub-problems, a novel kind of subproblem 3 is encountered in our problem. As shown in Fig 2, the spatial point ***p***_1_ moves along the axis ***ξ***_1_ by a distance *θ*_1_ to the given point ***p***_2_, the distance between position vectors ***p***_2_ and ***p***_3_ is given as *δ*. The unknown parameter *θ*_1_ can be formulated as

$$\text{\textbackslash{} }\left\{ \begin{array}{c} \theta_{\text{1}}=\left\|v\right\|\cos\theta_{\text{0}}+\delta^{\prime }\\ v=p_{\text{3}}-p_{\text{1}},\cos\theta_{\text{0}}=\left(\frac{\xi^{\text{T}}v}{\left\|v\right\|}\right)\\ {\delta^{\prime }}^{\text{2}}={\left(\left\|v\right\|\cos\theta_{\text{0}}\right)}^{\text{2}}-\left({\left\|v\right\|}^{\text{2}}-\delta^{\text{2}}\right) \end{array}\right.\;$$ (3)

where if *δ*^**′**2^ = 0, there will be one solution for subproblem 3, and vector ***p***_3_ – ***p***_1_ is perpendicular to vector ***ξ***_1_; else if *δ* ^**′**2^ > 0, two solutions exist while position vectors ***p***_2_ and ${p^{\prime }}_{\text{2}}$ lie on opposite sides of the perpendicular line to vector ***ξ***_1_. Otherwise, no solution exists. In particular, when point ***p***_3_ is on axis ***ξ***_1_, then *θ*_1_ = (***p***_3_ – ***p***_1_)^T^***ξ***_1_ – *δ*.

### 2.1. Structure of the 5PUS-RPUR parallel robot

**Fig 3:**
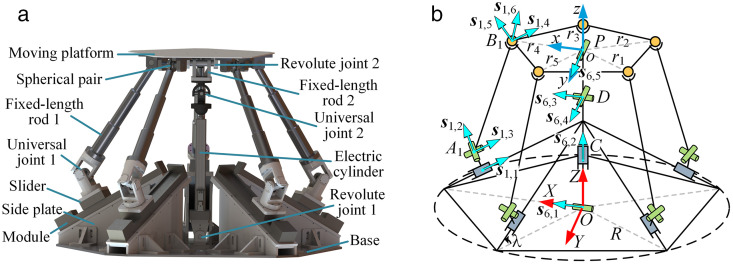
Virtual prototype and kinematic screw system of the 5PUS-RPUR parallel robot. (a) Virtual prototype. (b) Kinematic screw system.

The virtual prototype of the 5PUS-RPUR parallel robot is shown in Fig. 3a, which consists of a base, a moving platform, and two types of serial PUS and RPUR chains. The PUS chain contains a prismatic joint (P) comprised of a module, a side plate and a slider, a universal joint (U1) and a spherical joint (S), where U and S are connected by the fixed-length rod. The RPUR chain consists of two revolute joints (R), a prismatic joint composed of an electric cylinder and a push rod, a universal joint (U2) and a fixed-length rod, where the first revolute joint (R1) is directly connected to the base and the second revolute joint (R2) is connected to the universal joint via the fixed-length rod.

As shown in Fig. 3b, the kinematic screws of each branch chain are established in the static coordinate system. The screw of the P joint in the PUS chain is along its motion direction, and the U joint is decomposed into two R joints, with the twists of the R joints along their rotation axis directions. The S joint is decomposed into three R joints, one along the direction of the fixed-length rod *A*_*i*_*B*_*i*_, and the other two are parallel to the *X* and *Y* axes of the static coordinate system when its *Z*-axis is rotated to align to the direction of *A*_*i*_*B*_*i*_. For the RPUR chain, the universal joint is decomposed into two R joints, with screw directions parallel to the *X* axis of the static coordinate system and the *Y* axis of the moving coordinate system. The screw directions of the other two R joints are both along their axial directions. The screw direction of the P joint is parallel to the *z*-axis direction.

### 2.2. Inverse displacement analysis of the PUS chain

In terms of the joint connection form of the PUS chain, the pose of the center point on the moving platform can be expressed as


$$g_{i}(\theta )=\prod_{j=\text{1}}^{\text{5}}\text{exp}\left({\hat{\xi }}_{i,j},\theta_{i,j}\right)\cdot g_{i}\left(\text{0}\right),i=\text{1}\sim \text{5}$$ (4)

where exp(·) is the product of exponential formula [26]. *i* and *j* are the indices for the kinematic chain and the joint within that chain, respectively. ***g***_*i*_(0) and ***g***_*i*_(*θ*) represent the initial and desired pose of the moving platform. *θ*_*i*,*j*_ and $\text{\textbackslash{} }{\hat{\xi }}_{i,j}\left(j=\text{1}\sim \text{6}\right)$ denote the displacement and the kinematic screw of each branch joint.

Since the posterior three joint axes of the PUS chain intersect with a point *B*_*i*_, the following formula can be obtained according to the principle of position invariance.


$$\text{ exp}({\hat{\xi }}_{i,\text{4}},\theta_{i,\text{4}})\cdot \text{exp}({\hat{\xi }}_{i,\text{5}},\theta_{i,\text{5}})\cdot \text{exp}({\hat{\xi }}_{i,\text{6}},\theta_{i,\text{6}})\cdot P_{Bi}=P_{Bi}$$ (5)

with ***P***_*Bi*_ = [***p***_*Bi*_ 1]^T^, ***p***_*Bi*_ denotes the position vector of the point *B*_*i*_.

Taking the right multiplication for Eq. (4) with the inverse matrix ${𝐠}_{\text{i}}^{-\text{1}}\left(\text{0}\right)$ and matrix ***P***_*Bi*_, Eq. (4) can be rewritten as


$$\text{\textbackslash{} }g_{i}(\theta )g_{i}^{-\text{1}}\left(\text{0}\right)P_{Bi}=\text{exp}({\hat{\xi }}_{i,\text{1}},\theta_{i,\text{1}})\cdot \text{exp}({\hat{\xi }}_{i,\text{2}},\theta_{i,\text{2}})\text{exp}({\hat{\xi }}_{i,\text{3}},\theta_{i,\text{3}})P_{Bi}$$ (6)

Similarly, due to the last two joints in the first three joints converging at a point *A*_*i*_, it can be obtained from the principle of position invariance.


$$\text{ exp}({\hat{\xi }}_{i,\text{2}},\theta_{i,\text{2}})\cdot \text{exp}({\hat{\xi }}_{i,\text{3}},\theta_{i,\text{3}})\cdot P_{Ai}=P_{Ai}$$ (7)

with ***P***_*Ai*_ = [***p***_*Ai*_ 1]^T^, ***p***_*Ai*_ denotes the position vector of the point *A*_*i*_.

Taking the left dot product of Eq. (7) with $\text{ exp}(-{\hat{\xi }}_{i,\text{1}},\theta_{i,\text{1}})$, we get


$$\text{exp}(-{\hat{\xi }}_{i,\text{1}},\theta_{i,\text{1}})g_{i}(\theta )g_{i}^{-\text{1}}\left(\text{0}\right)P_{Bi}=\text{exp}({\hat{\xi }}_{i,\text{2}},\theta_{i,\text{2}})\text{exp}({\hat{\xi }}_{i,\text{3}},\theta_{i,\text{3}})P_{Bi}$$ (8)

Subtracting the matrix ***P***_*Ai*_ on both sides of Eq. (8), we obtain


$$\text{exp}(-{\hat{\xi }}_{i,\text{1}},\theta_{i,\text{1}})g_{i}(\theta )g_{i}^{-\text{1}}\left(\text{0}\right)P_{Bi}-P_{Ai}=\text{exp}({\hat{\xi }}_{i,\text{2}},\theta_{i,\text{2}})\text{exp}({\hat{\xi }}_{i,\text{3}},\theta_{i,\text{3}})\left(P_{Bi}-P_{Ai}\right)$$ (9)

According to the principle of distance invariance [27], it is easy to obtain


$$\left|P_{Bi}-P_{Ai}\right|=\left|\text{exp}({\hat{\xi }}_{i,\text{2}},\theta_{i,\text{2}})\text{exp}({\hat{\xi }}_{i,\text{3}},\theta_{i,\text{3}})\left(P_{Bi}-P_{Ai}\right)\right|$$ (10)

Substituting Eq. (10) into Eq. (9) shows that it corresponds to PK subproblem 3, and its parameters are expressed as follows.


$$\text{\textbackslash{} }\left\{ \begin{array}{c} \hat{\xi }=-{\hat{\xi }}_{i,\text{1}}^{\text{T}},p_{\text{1}}=g_{i}(\theta )g_{i}^{-\text{1}}\left(\text{0}\right)P_{Bi}\\ v=P_{Ai}-p_{\text{1}},\delta^{\text{2}}={\left\|P_{Bi}-P_{Ai}\right\|}^{\text{2}} \end{array}\right.\;$$ (11)

It is worth noting that the prismatic pair can be regarded as the revolute pair of the rotational axis at infinity. Substituting the parameters into PK subproblem 3, the analytical expression of *θ*_*i*,1_ can be derived.

On the basis of the known *θ*_*i*,1_, Eq. (9) can be regarded as PK subproblem 2, and the corresponding subproblem parameters are expressed as follows.


$$\left\{ \begin{array}{c} \xi_{\text{1}}={\hat{\xi }}_{i,\text{2}},\;\xi_{\text{2}}={\hat{\xi }}_{i,\text{3}},u=P_{Bi}-P_{Ai}\\ v=\text{exp}(-{\hat{\xi }}_{i,\text{1}},\theta_{i,\text{1}})g_{i}(\theta )g_{i}^{-\text{1}}\left(\text{0}\right)P_{Bi}-P_{Ai} \end{array}\right.\;$$ (12)

where the analytical expressions for *θ*_*i*,2_ and *θ*_*i*,3_ can be solved from the PK subproblem 2.

Substituting the equation $\text{exp}({\hat{\xi }}_{i,\text{6}},\theta_{i,\text{6}})P_{Ai}=P_{Ai}$ into Eq. (4), which can be rewritten as


$$\begin{array}{c} \text{exp}({\hat{\xi }}_{i,\text{4}},\theta_{i,\text{4}})\text{exp}({\hat{\xi }}_{i,\text{5}},\theta_{i,\text{5}})\left(P_{Bi}-P_{Ai}\right)=\text{exp}(-{\hat{\xi }}_{i,\text{3}},\theta_{i,\text{3}})\\ \text{exp}(-{\hat{\xi }}_{i,\text{2}},\theta_{i,\text{2}})\text{exp}(-{\hat{\xi }}_{i,\text{1}},\theta_{i,\text{1}})g_{i}(\theta )g_{i}^{-\text{1}}\left(\text{0}\right)\left(P_{Bi}-P_{Ai}\right) \end{array}$$ (13)

where the vector ***P***_*Ai*_ should be selected on the axis ${\hat{\xi }}_{i,\text{6}}$ and not on the axes ${\hat{\xi }}_{i,\text{4}}$ and ${\hat{\xi }}_{i,\text{5}}$. On the basis of the known ${\hat{\xi }}_{i,\text{1}}$, ${\hat{\xi }}_{i,\text{2}}$ and ${\hat{\xi }}_{i,\text{3}}$, the analytical expressions of *θ*_*i*,4_ and *θ*_*i*,5_ can be solved by using the inverse solution formula of PK subproblem 2.

Multiplying Eq. (5) on the left by $\text{exp}(-{\hat{\xi }}_{i,\text{5}},\theta_{i,\text{5}}cdots\text{exp}(-{\hat{\xi }}_{i,\text{1}},\theta_{i,\text{1}})$ and on the right by ${𝐠}_{\text{i}}^{-\text{1}}\left(\text{0}\right)$·^*A*^***p***, we can obtain


$$\text{exp}({\hat{\xi }}_{i,\text{6}},\theta_{i,\text{6}})\cdot^{A}p=\text{exp}(-{\hat{\xi }}_{i,\text{5}},\theta_{i,\text{5}})\cdots \text{exp}(-{\hat{\xi }}_{i,\text{1}},\theta_{i,\text{1}})\cdot g_{i}(\theta )\cdot g_{i}^{-\text{1}}\left(\text{0}\right)\cdot^{A}P$$ (14)

where ^*A*^***P*** = [^*A*^***p*** 1]^T^, ^*A*^***p*** is the position vector of any spatial point not on the rotational axis. The analytical expression of *θ*_*i*,6_ can be solved by using the inverse solution formula of PK subproblem 1.

### 2.3. Inverse displacement analysis of the RPUR chain

In terms of the structural form of the RPUR chain, the pose of the center point on the moving platform can be expressed as


$$g_{i}(\theta )=\text{exp}\left({\hat{\xi }}_{i,\text{1}},\theta_{i,\text{1}}\right)\cdot \text{exp}\left({\hat{\xi }}_{i,\text{2}},\theta_{i,\text{2}}\right)\cdot \text{exp}\left({\hat{\xi }}_{i,\text{3}},\theta_{i,\text{3}}\right)\cdot \text{exp}\left({\hat{\xi }}_{i,\text{4}},\theta_{i,\text{4}}\right)\cdot \text{exp}\left({\hat{\xi }}_{i,\text{5}},\theta_{i,\text{5}}\right)\cdot g_{i}\left(\text{0}\right),i=6$$ (15)

where $\text{\textbackslash{} }{\hat{\xi }}_{i,j}$ and *θ*_*i*, *j*_ (*j* = 1 ~ 5) represent the joint kinematic screw and joint displacement of the RPRU chain.

Expanding Eq. (15) and taking the first-row matrix from both sides, we obtain


$$\left[\begin{array}{cccc} \text{cos}(\beta )\text{cos}(\gamma ) & -\text{cos}(\beta )\text{sin}(\gamma ) & \text{sin}(\beta ) & x \end{array}]=[\begin{array}{cccc} \text{cos}\left(\theta_{i,\text{4}}+\theta_{i,\text{5}}\right) & \text{0} & \text{sin}\left(\theta_{i,\text{4}}+\theta_{i,\text{5}}\right) & \text{sin}\left(\theta_{i,\text{4}}\right)h_{\text{0}} \end{array}\right]$$ (16)

with $g_{i}(\theta )=[\begin{array}{cc} Rx(\alpha )Ry(\beta )Rz(\gamma ) & p\\ 0_{\text{1}\times \text{3}} & \text{1} \end{array}]$, $p=[\begin{array}{ccc} x & y & z \end{array}]\text{T}$,

where *α*, *β* and *γ* represent the Euler rotation angles of the moving platform around the *x*, *y* and *z* axes of the moving coordinate system. ***p*** denotes the position vector of the reference point on the moving platform. *h*_0_ is the distance from the center of U2 to the origin of the moving coordinate system. According to Eq. (16), *γ *= 0*.* This indicates that the 5PUS-RPUR parallel robot has the motion characteristic with three-dimensional positional movement and two-dimensional Eulerian angular rotation.

In terms of Eq. (16), the relationship between joint angle displacements *θ*_*i*,4_ and *θ*_*i*,5_ as well as the pose of the moving platform can be obtained.


$$\left\{ \begin{array}{c} \theta_{i,\text{4}}=\text{arcsin}\left(\frac{x}{h_{\text{0}}}\right)\\ \theta_{i,\text{5}}=\beta -\theta_{i\text{,4}} \end{array}\right.\;$$ (17)

where the expressions of joint angles *θ*_*i*,4_ and *θ*_*i*,5_ are obtained using the elimination method. Taking the right multiplication for both sides of Eq. (15) with the vector ***P***_*D*_, it can be obtained based on the principle of distance invariance.


$$\text{exp}({\hat{\xi }}_{i,\text{1}},\theta_{i,\text{1}})\cdot \text{exp}({\hat{\xi }}_{i,\text{2}},\theta_{i,\text{2}})\cdot P_{D}=g_{i}(\theta )\cdot g_{i}^{-\text{1}}\left(\text{0}\right)\cdot \text{exp}({\hat{\xi }}_{i,\text{5}},-\theta_{i,\text{5}})\cdot \text{exp}({\hat{\xi }}_{i,\text{4}},-\theta_{i,\text{4}})\cdot P_{D}$$ (18)

where ***P***_*D*_ denotes the position vector of the center of universal joint 2.

Due to the distance from the center of U to the origin of the static coordinate system is not affected by R2, the joint displacement *θ*_*i*,2_ can be solved by PK subproblem 3, and the subproblem parameters are expressed as


$$\left\{ \begin{array}{c} p_{\text{1}}=p_{\text{U}},p_{\text{3}}=p_{\text{O}},\;\xi ={\hat{\xi }}_{i,\text{2}}\text{,}\\ \delta =\left\|g_{i}(\theta )/g_{i}\left(\text{0}\right)\text{exp}({\hat{\xi }}_{i,\text{5}},-\theta_{i,\text{5}})\text{exp}({\hat{\xi }}_{i,\text{4}},-\theta_{i,\text{4}})P_{D}-P_{\text{O}}\right\| \end{array}\right.\;$$ (19)

where ***P***_o_ = [***p***_o_ 1]^T^, ***p***_o_ is the position vector of the origin of the moving coordinate system.

On the basis of the known joint displacements *θ*_*i*,2_, *θ*_*i*,4_ and *θ*_*i*,5_, the analytical expression of the joint angle *θ*_*i*,1_ can be obtained by converting Eq. (18) to PK subproblem 1, and the subproblem parameters are expressed as


$$\text{\textbackslash{} }\left\{ \begin{array}{c} r=p_{O},\;\hat{\xi }={\hat{\xi }}_{i,\text{1}},\;P_{1}=\text{exp}({\hat{\xi }}_{i,\text{2}},\theta_{i,\text{2}})\cdot P_{D},\\ P_{\text{2}}=g_{i}(\theta )/g_{i}\left(\text{0}\right)\cdot \text{exp}({\hat{\xi }}_{i,\text{5}},-\theta_{i,\text{5}})\cdot \text{exp}({\hat{\xi }}_{i,\text{4}},-\theta_{i,\text{4}})\cdot P_{D} \end{array}\right.\;$$ (20)

where ***p***_1_ and ***p***_2_ are matrices consisting of the first three columns of elements of matrices ***P***_1_ and ***P***_2_. ***P***_*D*_ = [***p***_*D*_ 1]^T^, ***p***_*D*_ is the position vector of the U center in the RPRU chain.

Likewise, the explicit expressions of joint displacements *θ*_*i*,3_ can be analyzed by Eq. (15) equivalent to PK subproblem 1, and the subproblem parameters are expressed as


$$\left\{ \begin{array}{c} r=p_{D},\hat{\xi }={\hat{\xi }}_{i,\text{3}},p_{\text{1}}=p_{O}\\ p_{\text{2}}=\text{exp}({\hat{\xi }}_{i,\text{2}},\theta_{i,\text{2}})\cdot \text{exp}({\hat{\xi }}_{i,\text{1}},\theta_{i,\text{1}})\cdot g_{i}(\theta )\cdot g_{i}^{-\text{1}}\left(\text{0}\right)\cdot \text{exp}({\hat{\xi }}_{i,\text{5}},-\theta_{i,\text{5}})\cdot \text{exp}({\hat{\xi }}_{i,\text{4}},-\theta_{i,\text{4}})P_{O} \end{array}\right.\;$$ (21)

At this point, the analytical formulas of each branched chain joint have been obtained. It should be noted that there will be generally two solutions in the PK subproblems 1 and 2, and the sum value of the two solutions is π or -π. The correct solution can be obtained by setting the constraint conditions. The PK subproblem 3 yields two solutions, and the solution with the smaller absolute displacement is correct.

## 3. Error and sensitivity analysis

### 3.1. Error modeling of the PUS chain

**Fig 4:**
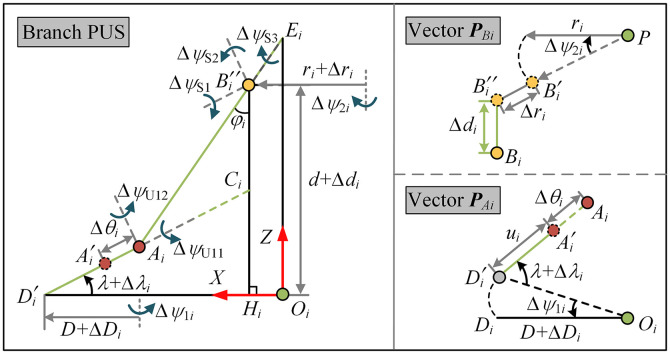
Structure diagram of branched PUS with errors.

A closed-loop vector method is used for the error analysis of the 5PUS-RPUR parallel robot. Considering the manufacturing and assembly errors, the error mapping model of the PUS chain is analyzed. On the basis of Fig. 3(b), the geometric errors in the PUS chain are described in Fig. 4.

From the analysis of the inverse kinematics, the pose mapping relationship between the moving platform and the actuator joints only requires the initial and final pose of the moving platform, and the position vector of the U center point *A*_*i*_ and the S center point *B*_*i*_. The main factors affecting the position vector of point *A* include the module position deviation ∆*ψ*_1*i*_, the side plate angle deviation ∆*λ*_*i*_, the actuator zero-point error ∆*θ*_*i*_, and the manufacturing error of the base length ∆*D*_*i*_. The position error of the vector *O*_*i*_*B*_*i*_ is affected by the assembly error ∆*ψ*_2*i*_ of the spherical pair and the manufacturing error ∆*d*_*i*_ of the bearing. Considering the above conversion errors, the forward kinematics of the PUS chain can be expressed as


$$\Delta g(\theta )=\prod_{j=\text{1}}^{\text{6}}\text{exp}\left(\Delta {\hat{\xi }}_{i,j},\Delta \theta_{i,j}\right)\cdot \Delta g\left(\text{0}\right),\;i=\text{1}\sim \text{5}$$ (22)

$$\text{\textbackslash{} }\left\{ \begin{array}{c} \Delta {\hat{\xi }}_{i,\text{1}}={\left[\text{000},\Delta s_{i\text{,1}}\right]}^{\text{T}},\Delta s_{i\text{,1}}=R_{i\text{,1}}{\left[\text{001}\right]}^{\text{T}},R_{i\text{,1}}=Rz\left(\text{2}\left(i-\text{1}\right)\text{π}/\text{5}\right)Ry(\lambda +\Delta \lambda -\text{π}/\text{2})\\ \Delta {\hat{\xi }}_{i,\text{2}}={\left[\Delta s_{i\text{,2}}{\text{0}P}_{Ai}\times s_{i\text{,2}}\right]}^{\text{T}},\Delta s_{i\text{,2}}=R_{i\text{,2}}e_{y},R_{i\text{,2}}=Rz\left(\text{2}\left(i-\text{1}\right)\text{π}/\text{5}\right)Ry(-\varphi_{i})Ry(\Delta \lambda )\\ \Delta {\hat{\xi }}_{i,\text{3}}={\left[\Delta s_{i\text{,3}}{\text{0}P}_{Ai}\times s_{i\text{,3}}\right]}^{\text{T}},\Delta s_{i\text{,3}}=R_{i\text{,3}}e_{x},R_{i\text{,3}}=R_{i\text{,2}}Ry(\Delta \psi_{\text{U11}})\\ \Delta {\hat{\xi }}_{i,\text{4}}={\left[\Delta s_{i\text{,4}}{0P}_{Bi}\times \Delta s_{i\text{,4}}\right]}^{\text{T}},\Delta s_{i\text{,4}}=R_{i\text{,4}}e_{x},R_{i\text{,4}}=R_{i\text{,3}}Rx(\Delta \psi_{\text{U12}})\\ \Delta {\hat{\xi }}_{i,\text{5}}={\left[\Delta s_{i\text{,5}}{\text{0}P}_{Bi}\times \Delta s_{i\text{,5}}\right]}^{\text{T}},\Delta s_{i\text{,5}}=R_{i\text{,5}}e_{y},R_{i\text{,5}}=R_{i\text{,4}}Ry(\Delta S_{\text{11}})\\ \Delta {\hat{\xi }}_{i,\text{6}}={\left[\Delta s_{i\text{,6}}{\text{0}P}_{Bi}\times \Delta s_{i\text{,6}}\right]}^{\text{T}},\Delta s_{i\text{,6}}=R_{i\text{,6}}e_{z},R_{i\text{,6}}=R_{i\text{,5}}Rx(\Delta S_{\text{12}}) \end{array}\right.\;$$ (22a)

$$\begin{array}{c} \left\{ \begin{array}{c} {0P}_{Ai}=O_{i}{D^{\prime }}_{i}+{D^{\prime }}_{i}A_{i},{0P}_{Bi}=O_{i}{B^{\prime }}_{i}+{B^{\prime }}_{i}B_{i}\\ O_{i}{D^{\prime }}_{i}=Rz\left(\text{2}\left(i-\text{1}\right)\text{π}\;/\text{5}+\Delta \psi_{1i}\right)D_{i},D_{i}={\left[D+\Delta D_{i}\;0\;0\right]}^{\text{T}}\\ {D^{\prime }}_{i}A_{i}=\left(u_{i}+\Delta \theta_{i}\right)\left[Rz\left(\text{2}\left(i-\text{1}\right)\text{π}\;/\text{5}+\Delta \psi_{1i}\right)\left(Ry\left(-\lambda -\Delta \lambda_{i}\right)\right)e_{z}\right]\\ O_{i}{B^{\prime }}_{i}=\Delta R^{N}B_{i}+\Delta P,\Delta R=R_{x}(\Delta \alpha )R_{y}(\Delta \beta ){,}^{N}B_{i}={\left[0\;0\;\Delta d_{i}\right]}^{\text{T}}\\ {B^{\prime }}_{i}B_{i}=Rz\left(\text{2}\left(i-\text{1}\right)\text{π}\;/\text{5}+\Delta \psi_{2i}\right)\left(P{B^{\prime }}_{i}+{B^{\prime \prime }}_{i}B_{i}\right)\\ P{B^{\prime }}_{i}={\left[r_{i}\;0\;0\right]}^{\text{T}},{B^{\prime \prime }}_{i}B_{i}={\left[\Delta r_{i}\;0\;\Delta d_{i}\right]}^{\text{T}},\Delta P={\left[\Delta x\;\Delta y\;\Delta z\right]}^{\text{T}} \end{array}\right.\; \end{array}$$ (22b)

where $\Delta {\hat{\xi }}_{i,j}$ denotes the screw axis of the *j*-th joint in the *i*-th PUS limb, considering geometric errors. $\Delta {\hat{\theta }}_{\text{i}\text{,}\text{j}}$

represents the actual joint displacement, inclusive of geometric errors. ***e***_*x*_, ***e***_*y*_ and ***e***_*z*_ are the unit vectors along the *X*, *Y*, and *Z* axes of the fixed coordinate frame. ^0^***P***_*Ai*_ and ^0^***P***_*Bi*_ denote the position vectors of the center points of the U and S at the initial pose. ∆***P*** and ∆***R*** represent the initial position and posture error of the moving platform, respectively. *φ*_*i*_ is the nominal angle between the constant-length link *A*_*i*_*B*_*i*_ and the Z-axis of the fixed frame. *r*_*i*_ and *d* are the distance of point *B*_*i*_ from the origin of the moving coordinates and the distance of point *B* from the *XOY* plane. The parameters with manufacturing error include ∆*r*_*i*_, ∆*D*_*i*_ and ∆*d*_*i*_, and the parameters with assembly error include ∆*ψ*_1*i*_, ∆*ψ*_2*i*_ and ∆*λ*_*i*_. By replacing the vector points ***P***_*Ai*_ and ***P***_*Bi*_ in the inverse kinematics of the PUS chain with the vectors ^0^***P***_*Ai*_ and ^0^***P***_*Bi*_.

### 3.2. Error modeling of the RPUR chain

**Fig 5:**
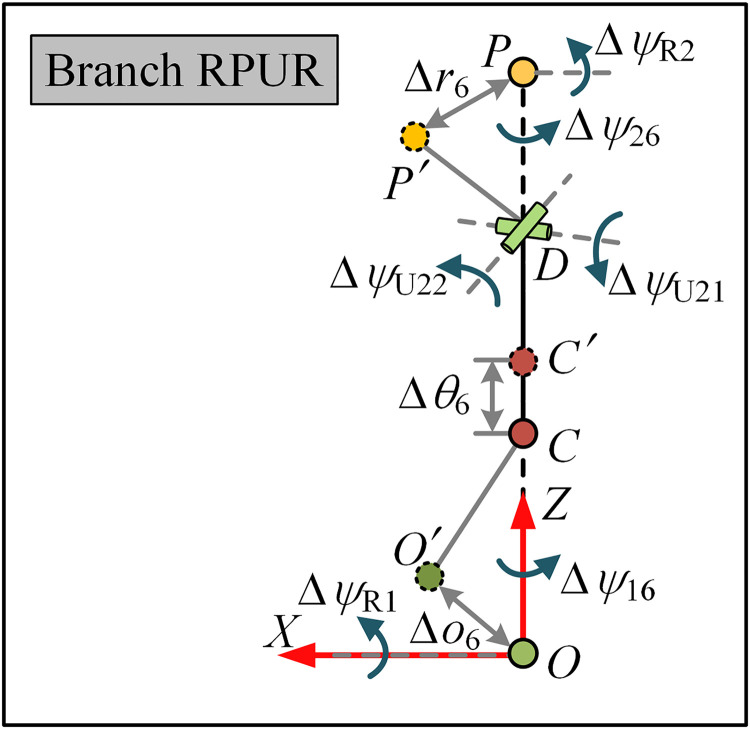
Structure diagram of branched RPUR with errors.


The geometric errors in the RPUR chain are described in Fig 5, including the assembly pose error of R1, the initial position error of the electric cylinder, the angular error of U2, the assembly pose error of R2, and the initial pose error of the moving platform. The relationship between these geometric source errors and the pose of the moving platform is established using forward kinematics and can be formulated as

$$\;\Delta g(\theta )=\prod_{j=\text{1}}^{\text{5}}\text{exp}\left(\Delta {\hat{\xi }}_{i,j},\Delta \theta_{i,j}\right)\cdot \Delta g\left(\text{0}\right),i=6$$ (23)

$$\text{\textbackslash{} }\left\{ \begin{array}{c} \Delta \hat{\xi }\text{6},\text{1}=\left[\Delta si,1\;0P_{o}\times \Delta s_{i\text{,1}}\right]\text{T},\Delta s_{i\text{,1}}=R_{\text{6,1}}\left[\text{100}\right]\text{T},R_{\text{6,1}}=Rz(\Delta \psi_{\text{16}})\\ \Delta {\hat{\xi }}_{\text{6},\text{2}}=\left[\text{000}\Delta s_{\text{6,2}}\right]\text{T},\Delta s_{\text{6,2}}=R_{\text{6,2}}\left[\text{001}\right]\text{T},R_{\text{6,2}}=R_{\text{6,1}}Rx\left(\Delta \psi_{\text{R1}}\right)\\ \Delta {\hat{\xi }}_{\text{6},\text{3}}=\left[\Delta s_{\text{6},\text{3}}{\text{0}P}_{D}\times s_{\text{6},\text{3}}\right]\text{T},\Delta s_{\text{6,3}}=R_{\text{6,3}}\left[\text{100}\right]\text{T},R_{\text{6,3}}=R_{\text{6,2}}Ry\left(\Delta \psi_{\text{U22}}\right)\\ \Delta {\hat{\xi }}_{\text{6},\text{4}}=\left[\Delta s_{\text{6},\text{4}}{\text{0}P}_{D}\times s_{\text{6},\text{4}}\right]\text{T},\Delta s_{\text{6,4}}=R_{\text{6,4}}[\text{0}\;\text{1}\text{0}]\text{T},R_{\text{6,4}}=R_{\text{6,3}}Rx\left(\Delta \psi_{\text{U21}}\right)\\ \Delta {\hat{\xi }}_{\text{6},\text{5}}=\left[\Delta s_{\text{6},\text{5}}{\text{0}P}_{P^{\prime }}\times s_{\text{6},\text{5}}\right]\text{T},\Delta s_{\text{6,5}}=R_{\text{6,5}}[\text{0}\text{10}]\text{T},R_{\text{6,5}}=R_{\text{6,5}}Rz\left(\Delta \psi_{\text{26}}\right) \end{array}\right.\;$$ (23a)

$$\left\{ \begin{array}{c} {0P}_{D}={0P}_{O^{\prime }}+O^{\prime }C+CD,{0P}_{P^{\prime }}=Rx\left(\Delta \psi_{\text{R2}}\right)Rz\left(\Delta \psi_{26}\right){\left[\Delta r_{6}\;0\;0\right]}^{T}\\ {0P}_{O^{\prime }}=Rz\left(\Delta \psi_{16}\right){[}^{\Delta },O^{\prime }C=Rx\left(\Delta \psi_{r1}\right){\left[0\;0\;l_{c}\right]}^{T},CD={\left[0\;0\;l_{d}+\Delta \theta_{6}\right]}^{T} \end{array}\right.\;$$ (23b)

where ^0^***P***_*O′*_, ^0^***P***_*D*_ and ^0^***P***_*P′*_ denote the position vectors of the center points of the *O′*, *D* and *P′* at the initial pose. *l*_*c*_ is the center distance from the electric cylinder to U2, and *l*_*d*_ is the center distance from push rod to the origin. Δ*θ*_6_ represents the zero-position error of the electric cylinder, and ∆*ψ*_r1_ represents the initial deviation of R1.

### 3.3. Sensitivity analysis

As stated above, there are 69 error sources affecting the moving platform pose accuracy of the parallel robot. The individual identification of each source is complex, tedious, and computationally expensive. Therefore, a geometric error sensitivity model is established to screen out errors that have a minor impact on the moving platform pose. The mapping between the geometric errors and the moving platform’s pose can be obtained from Eqs. (22) and (23). Taking the degree of freedom of the PUS chain in the *x*-direction as an example, the sensitivity index is established as follows


$$\text{\textbackslash{} }\eta_{xi}=\frac{\partial x}{\partial q_{pi}}$$ (24)

where *q*_*pi*_ denotes the vector of geometric error sources affecting the position *x*, namely *q*_*pi*_: {Δ*ψ*_1*i*_, Δ*ψ*_2*i*_, Δ*λ*_*i*_, Δ*D*_*i*_, Δ*r*_*i*_, Δ*d*_*i*_, Δ*θ*_*i*_}. Additionally, the other error vectors include *q*_*ωi*_: {Δ*ψ*_1*i*_, Δ*ψ*_2*i*_, Δ*λ*_*i*_, Δ^1^*ψ*_U11_, Δ^1^*ψ*_U12_, Δ*ψ*_S11_, Δ*ψ*_S12_,Δ*ψ*_S13_}, *q*_*p*6_: {Δ*ψ*_R1_, Δ*ψ*_16_, Δ*o*_6_, Δ*θ*_6_, Δ*ψ*_R2_, Δ*ψ*_16_, Δ*r*_6_}, and *q*_*ω*6_: {Δ*ψ*_R1_, Δ*ψ*_16_, Δ*ψ*_R2_, Δ*ψ*_26_, Δ*ψ*_U21_, Δ*ψ*_U22_}.

In order to visually analyze the workspace, the Tilt-and-Torsion (T&T) angle containing azimuth *ϕ*_*P*_, tilt *θ*_*P*_ and torsion *ψ*_*P*_ is used to describe the posture change of the parallel robot [28]. The rotation matrix of the T&T angle can be expressed as


$${\text{\textbackslash{} }}^{TT}R\left(\phi_{P},\theta_{P},0\right)=R_{z}\left(\phi_{P}\right)R_{y}\left(\theta_{P}\right)R_{z}\left(-\phi_{P}\right)R_{z}(0)$$ (25)

Due to the degree of freedom property of the 5PUS-RPUR parallel robot, the Euler angle γ of rotation of the moving platform around the *z*-axis is equal to 0. The Euler angles are converted into the T&T angle form, which can be expressed as


$$\text{\textbackslash{} }\left\{ \begin{array}{c} \alpha =\text{asin}\left(-\text{sin }\phi_{P}\text{ sin }\theta_{P}\right)\\ \beta =\text{asin}\left(\text{cos }\phi_{P}\text{ sin }\theta_{P}\right) \end{array}\right.\;$$ (26)

The workspace of the 5PUS-RPUR parallel robot is limited by the range of the actuators, the mechanical limit of passive joints and the interference between links. The mathematical expression of the workspace is


$$W=\{\;\;\left(x,y,z,\phi_{P},\theta_{P}\right)\in R^{5}|f\left(x,y,z,\phi_{P},\theta_{P}\right)\leq 0\}\;$$ (27)

where ***f*** (⋅) denotes the constraints, which include rod length, corner and link interference constraints. *x*, *y*, *z* and *ϕ*_*P*_, *θ*_*P*_ are the position and posture parameters of the moving platform respectively. For the 5PUS-RPUR parallel robot, the *x* limit range is determined by the length of the fixed-length rod 2, the *y* limit range is the sum of the parameter *d* and the maximum displacement of the actuator, and the *z* limit range is the maximum displacement of the actuator. While the azimuth angle *ϕ*_*P*_ is within [0, 2π] and the tilt angle *θ*_*P*_ is within [0, π/2]. Then, the above pose parameters are evenly divided, and the divided parameters are combined and substituted into the inverse kinematics of the PUS and RPUR chains to obtain the poses of the parallel robot’s components. After that, it is judged whether the poses satisfy the constraints; all valid poses satisfying the constraints constitute the reachable workspace of the parallel robot.

(1) Rod length constraints: *θ*_min_ ≤ *θ*_*i*_ ≤ *θ*_max_ (*i* = 1 ~ 5), *θ*_min_ and *θ*_max_ indicate the minimum and maximum displacements of the actuator joint.
(2) Corner constraints: *θ*_*Ui*_ = arccos[$\hat{L}$_*i*_·(***s***_*i*,2_ × ***s***_*i*,3_)]≤*θ*_*U*max_,

*θ*_*Si*_ = arccos($\hat{L}$_*i*_·***s***_*i*,6_)≤*θ*_*S*max_, *θ*_*U*max_ and *θ*_*S*max_ indicate the maximum rotation angle of the U and S pair.

(3) Link interference constraints: *d*_*R*_ ≥ *d*_*sa*_, *D*_*P*6_ ≥ *D*_1_ + *D*_2_, *d*_*sa*_ is the interference distance between the fixed-length rod 2 and the moving platform, which can be limited with the rotation angle of the R pair in the RPUR chain connected to the moving platform. Without loss of generality, the actuator joints of the RPUR and PUS chains are equated to spheres with diameters *D*_1_ and *D*_2_, respectively.

**Fig 6:**
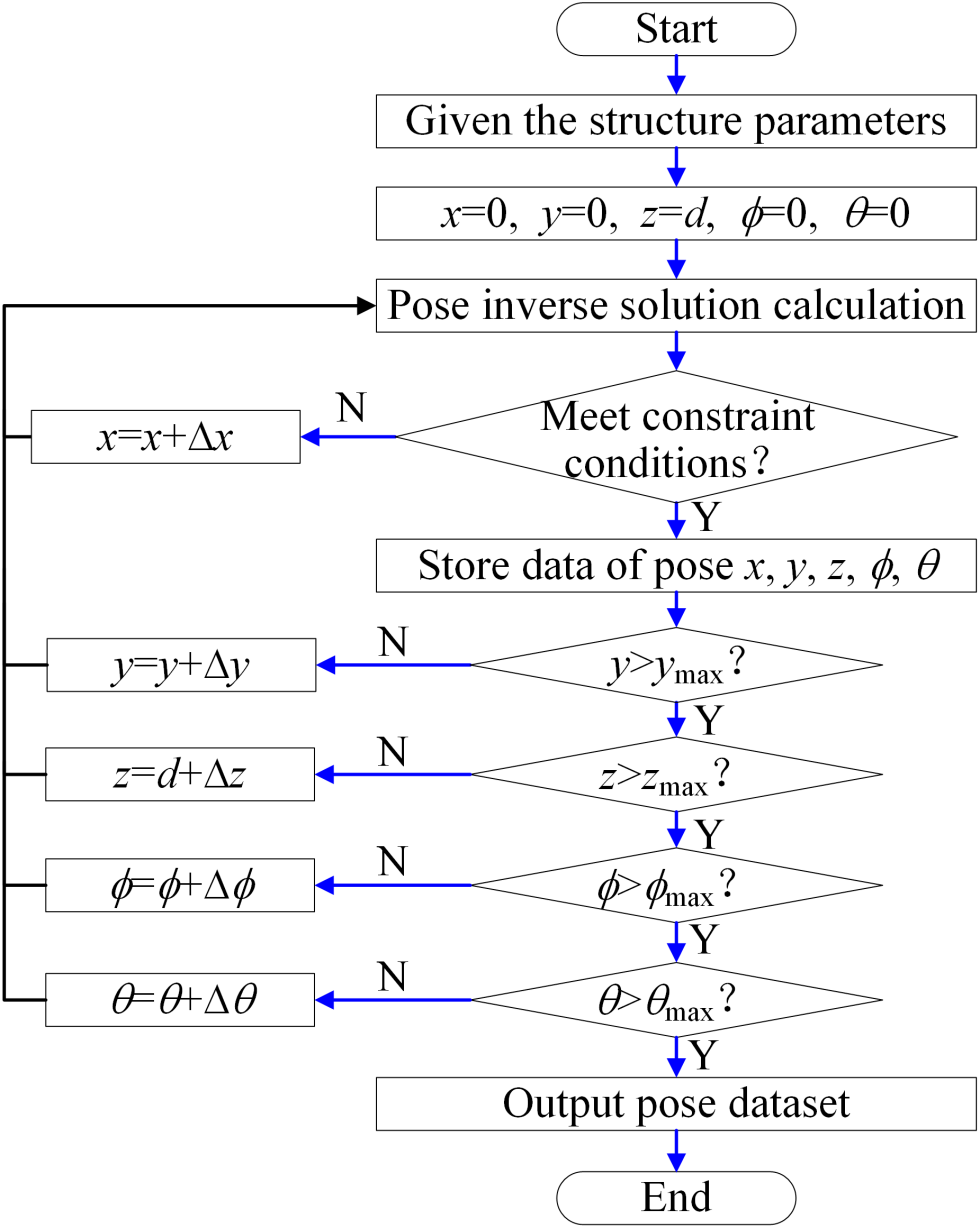
Flow diagram of the reachable workspace.

As shown in Fig. 6, the reachable workspace of the 5PUS-RPUR parallel robot is analyzed by the search method. First, the range of pose parameters *x*, *y*, *z* and *ϕ*_*P*_, *θ*_*P*_ are determined according to the structural characteristics, and the structure size of the parallel robot is given in Table 1. Next, the step size of pose parameters is set to Δ*x* = Δ*y* = Δ*z* = 0.01m and Δ*ϕ*_*P*_ = Δ*θ*_*P*_ = π/60 rad. Substitute the initial values of the moving platform’s pose parameters into the inverse kinematics of the parallel robot to obtain the poses of each component, and then iteratively check whether the current pose parameters of the moving platform satisfy the rod length, corner, and link interference constraints. If the constraints are satisfied, store the current poses; otherwise, discard the poses. Finally, the set of all poses satisfying the constraints is output, which represents the reachable workspace of the parallel robot’s moving platform.

**Table 1:** Size parameters of the 5PUS-RPUR parallel robot.

Parameter	Symbol	Value (unit)
Center distance from 1# S pair to movable platform	*r*_1_	200 mm
Center distance from 2# S pair to movable platform	*r*_2_	213.13 mm
Center distance from 3# S pair to movable platform	*r*_3_	225.60 mm
Center distance from 4# S pair to movable platform	*r*_4_	237.47 mm
Center distance from 5# S pair to movable platform	*r*_5_	248.78 mm
Center distance from the electric cylinder to the universal joint 2	*l*_*c*_	514 mm
Center distance from push rod to origin	*l*_*d*_	113 mm
Distance from the intersection of the side plate plane and the *X*-axis to the origin	*D*	1000 mm
Distance from point *B* to *XOY* plane	*d*	760 mm
Length of fixed-length rod 1	*l*_1_	600 mm
Length of fixed-length rod 2	*l*_2_	132 mm
Side plate angle	*λ*	30°

**Fig 7:**
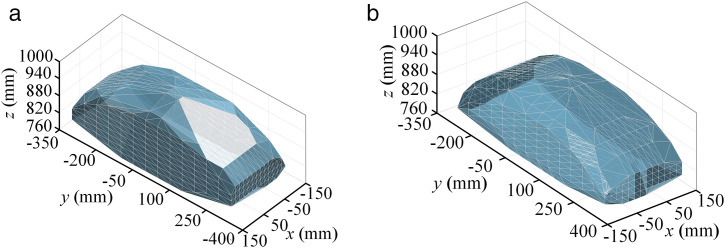
Reachable pose workspace of the 5PUS-RPUR parallel robot. **(a)** Position workspace (left view). **(b)** Position workspace (right view).

Following the steps in Fig 6 the parallel robot pose reachable workspace is obtained as shown in Fig 7. From the side view, the position workspace of the reference point is trapezoidal distribution, which is determined by the structure of the parallel robot. On the other hand, the posture workspace is basically enveloped in an entire circle, which can achieve flexible rotation within a limited range.

**Fig 8:**
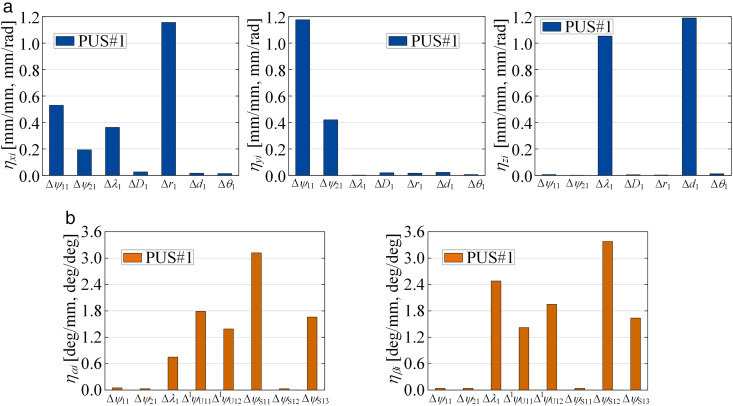
Mean of sensitivity indices of PUS chain throughout the reachable workspace. **(a)** Mean of position sensitivity indices of PUS chain. **(b)** Mean of posture sensitivity indices of PUS chain.

**Fig 9:**
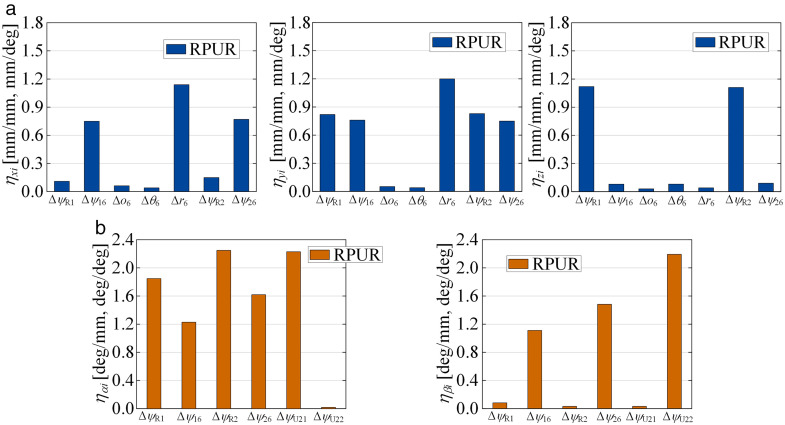
Mean of sensitivity indices of RPUR chain throughout the reachable workspace. **(a)** Mean of position sensitivity indices of PRUR chain. **(b)** Mean of posture sensitivity indices of PRUR chain.

Given the strong coupling and nonlinear characteristics inherent among the source errors in Eq. (24), deriving an independent analytical relationship between each error and the moving platform’s pose is exceedingly difficult. Therefore, a numerical method is employed to solve for the partial derivatives [29]. The mean sensitivity of each error source is calculated at different pose points based on the structural parameters mentioned above. The resulting histograms of the mean sensitivity for position and posture errors are shown in Figs 8 and 9, respectively. It can be observed that the robot’s moving platform pose is highly sensitive to angular variations within the geometric errors. The error sources with a minor impact on the moving platform’s position error, namely Δ*D*_*i*_, Δ*θ*_*i*_, Δ*o*_6_, and Δ*θ*_6_, are screened out to simplify the complexity and improve the efficiency of parameter identification.

## 4. Kinematic calibration

**Fig 10:**
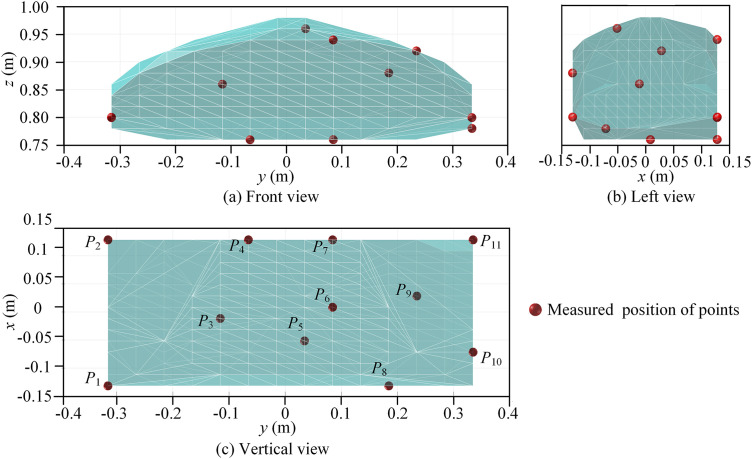
Distribution of measured points in the position workspace.

**Fig 11:**
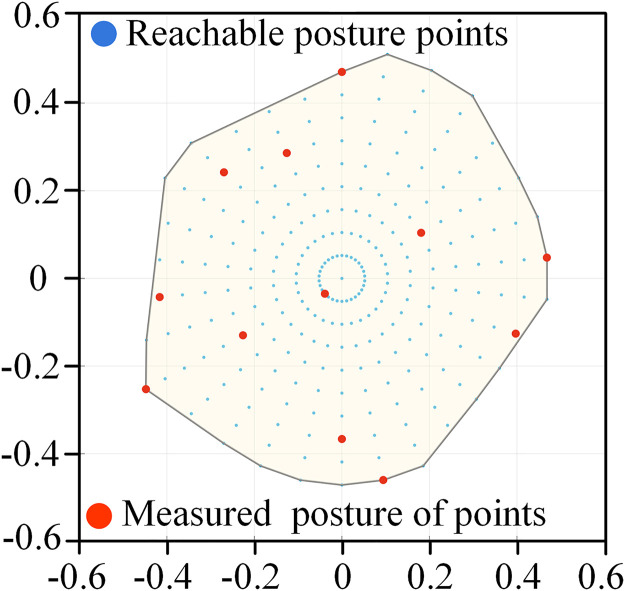
Distribution of posture measured points at the fixed position.

To ensure the effective identification of geometric source errors, the selection of measured points is crucial. The measured points should go through all controllable degrees of freedom. Moreover, it is believed that measuring enough poses is beneficial to increase the identification robustness [30]. However, in practical applications, a compromise must be struck between identification robustness and calibration efficiency. Research indicates that the number of identification equations should be at least twice the number of parameters to be identified [19]. Following a sensitivity analysis, 57 parameters have been identified for estimation, dictating a requirement for no fewer than 114 measurement poses. In light of this, 121 uniform measured points are selected within the prescribed workspace using a farthest point sampling algorithm [31]. This set includes 11 position points, with 11 different posture points selected at each of these fixed positions. The specific distribution is illustrated in Figs 10 and 11.

Fig 11 shows the distribution of measured posture points when the moving platform’s reference point is fixed at *P*_3_ = [−11.6, −115.4, 860] mm. This distribution is also generated using the farthest point sampling algorithm.

**Fig 12:**
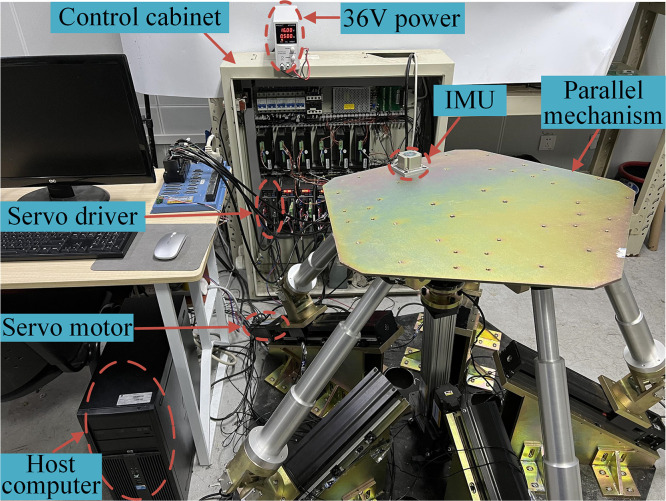
5PUS-RPUR parallel robot system.

To validate the kinematic calibration process of the 5PUS-RPUR parallel robot, the kinematic calibration experiment is carried out. The 5PUS-RPUR parallel robot system is built as shown in Fig 12, including a host computer, GTS motion control card, IMU sensor, servo motor with encoder and parallel mechanism body.

In the kinematic calibration experiment, the theoretical displacement of each actuator is obtained by applying theoretical pose measured points of the moving platform to the inverse kinematics. The motion command of the theoretical displacement is input in the host computer, and the pose variation of the moving platform is measured by IMU. Considering the manufacturing and assembly errors and the initial pose deviation of the moving platform, the IMU measured data is substituted into the error mapping model. It should be noted that the IMU cannot measure absolute poses. The joint displacement calculated by the first collected data is used as the reference, and the joint displacement obtained by other pose data minus the reference is used as the available data. Furthermore, exclusively considering the discrepancy in actuator displacement, derived from encoder and IMU data, while neglecting the coupling effects between kinematic chains, would inevitably introduce coupling errors and degrade the identification accuracy. Therefore, a comprehensive objective function is established by integrating the actuator displacement deviations with the pose error of the moving platform. This can be expressed as:


$$\text{\textbackslash{} }\left\{ \begin{array}{c} \text{min}\;f(X)=(1-\varpi_{1}-\varpi_{2})\sum_{i=1}^{6}\left\|\Delta \theta_{i}^{e}-\Delta \theta_{i}^{\text{IMU}}\right\|+\sum_{i=1}^{6}\left(\varpi_{1}\Delta E_{Pi}+\varpi_{2}\Delta E_{Ri}\right)(i=1\sim 6)\\ s.t.\;X\in R,R\subseteq U,\;l_{1}-\Delta l_{1}\leq \left\|P_{Ai}-P_{Bi}\right\|\leq l_{1}+\Delta l_{1},\;l_{2}-\Delta l_{2}\leq \left\|P_{D}-P_{P^{\prime }}\right\|\leq l_{2}+\Delta l_{2} \end{array}\right.\;$$ (28)

where Δ*E*_*pi*_ and Δ*E*_*ri*_ represent the Euclidean norms of the position and posture errors of the moving platform, respectively, as derived from the forward kinematics of each chain. The terms *ϖ*_1_ and *ϖ*_2_ are weighting factors that balance the contribution of the position and posture errors. It is important to note that to address the issue of dimensional inconsistency, all three sub-terms within *f*(***X***) have been normalized using min-max scaling [32]. ***X*** represents variables such as manufacturing error and assembly error, initial pose deviation and pose offset of moving platform. $\theta_{\text{i}}^{\text{e}}$ and $\theta_{\text{i}}^{\;\text{IMU}}$ indicate the actuator displacement measured by the encoder and obtained by substituting IMU data into the error mapping model. *i* denotes the branch number. *U* represents the basic space of decision variables, and *R* is a subset of *U*. The solution *X* satisfying the constraint condition is called the feasible solution, and the set *R* represents the set of feasible solutions. *l*_1_ and *l*_2_ denote the length of the fixed-length rod 1 and 2. Δ*l*_1_ and Δ*l*_2_ is the manufacturing error of the fixed-length rod 1 and 2 to be measured using a high-precision instrument. In the process of establishing the error model, only the vector position of the two ends of the fixed-length rod is described from the base and the moving platform, and the rod length error needs to be added to ensure the rationality of the model.

Genetic algorithm has the advantages of good generalization, strong robustness and global optimality in dealing with nonlinear constraints [33]. Due to it being time-consuming to deal with nonlinear constraints, Eq. (28) can be transformed into an unconstrained problem through a penalty function method.


$$\text{\textbackslash{} }\begin{array}{c} f_{P}(X)=f(X)+\sum_{i=1}^{5}G\left\{\text{max}\left[\left\|P_{Ai}-P_{Bi}\right\|-\left(l_{1}+\Delta l_{1}\right)\text{,0}\right]+\text{max}\left[\left(l_{1}-\Delta l_{1}\right)-\left\|P_{Ai}-P_{Bi}\right\|\text{,0}\right]\right\}\\ +H\left\{\text{max}\left[\left\|P_{D}-P_{P^{\prime }}\right\|-\left(l_{2}+\Delta l_{2}\right)\text{,0}\right]+\text{max}\left[\left(l_{2}-\Delta l_{2}\right)-\left\|P_{D}-P_{P^{\prime }}\right\|\text{,0}\right]\right\} \end{array}$$ (29)

where *G* and *H* are penalty factors, which are typically set to large positive values. According to the evolutionary mechanism of the genetic algorithm, the population will autonomously avoid solutions that do not satisfy the constraints during the evolutionary process. The optimal error parameters can be identified using genetic algorithms, and the errors of manufacturing, assembly and actuator initial positions can be compensated to improve the pose accuracy of the parallel robot.

The pose data of the moving platform and the displacement of the actuator measured by IMU and encoder are substituted into Eq. (30). The initialization parameters of genetic algorithm are as follows: population = 200, iteration number = 100, crossover probability = 0.8, mutation probability = 0.05. The optimization results are shown in Table 2 and Table 3.

**Table 2:** Optimization results of manufacturing and assembly errors in the PUS chain.

CN	∆*ψ*_1*i*_	∆*ψ*_2*i*_	∆*λ*_*i*_	∆*r*_*i*_	∆*d*_*i*_	∆*ψ*_*Ui*1_	∆*ψ*_*Ui*2_	∆*ψ*_*Si*1_	∆*ψ*_*Si*2_	∆*ψ*_*Si*3_
PUS#1	−0.08	0.14	0.076	−0.98	−1.11	−0.04	0.04	0.01	0.04	0.06
PUS#2	−0.06	0.09	0.081	−0.89	−1.04	0.03	0.07	−0.02	0.01	−0.02
PUS#3	0.09	−0.21	−0.071	0.88	0.94	−0.06	−0.06	0.03	−0.03	0.01
PUS#4	0.12	−0.24	0.049	−1.02	−0.96	0.06	0.07	−0.01	−0.03	−0.03
PUS#5	−0.11	0.18	0.062	1.06	0.87	0.05	−0.04	0.02	0.02	0.04

Note: Angle unit: °, length unit: mm, Chain number (CN).

**Table 3:** Optimization results of manufacturing and assembly errors in the RPUR chain.

Chain	∆*ψ*_*R*1_	∆*ψ*_16_	∆*o*_6_	∆*ψ*_*R*2_	∆*ψ*_26_	∆*ψ*_*U*21_	∆*ψ*_*U*22_
RPUR	0.08	−0.06	0.16	−0.07	0.03	0.08	−0.06

Note: Angle unit: °, length unit: mm, Chain number (CN).

After performing the kinematic calibration, the identified parameters are embedded into the control model, replacing the nominal ones. To evaluate the kinematic calibration experiment, the pose accuracy is analyzed using the pose points of another 36 test poses points before and after kinematic calibration. The pose points consist of 9 uniformly selected position points, with 4 posture points chosen at each position, corresponding to the maximum and minimum posture values. The corresponding data are presented in Table 4. The data processing for encoders and IMU sensors is embedded in the proprietary software, where the manufacturers have performed filtering, outlier removal, and smoothing of the measurement data.

**Table 4:** Dataset of measurement poses and actuator displacements.

Measured point	∆*x*	∆*y*	∆*z*	∆*α*	∆*β*	$\theta_{\text{1}}^{\text{e}}$	$\theta_{\text{2}}^{\text{e}}$	$\theta_{\text{3}}^{\text{e}}$	$\theta_{\text{4}}^{\text{e}}$	$\theta_{\text{5}}^{\text{e}}$
1	−131.18	335.3	760.33	−20.08	−21.50	233.96	−69.80	−221.88	−15.60	259.14
2	−131.20	335.3	760.36	−20.10	24.53	50.24	−122.26	−19.08	259.62	170.34
3	−131.14	335.1	760.40	21.98	−21.53	207.99	111.13	−143.52	−41.28	102.32
4	−131.12	335.1	760.36	21.88	24.53	76.52	15.74	123.86	85.40	74.24
5	−131.15	10.29	920.38	−20.97	−22.33	376.63	167.28	−6.60	82.35	421.46
6	−131.16	10.27	920.30	−20.93	25.39	153.53	104.11	247.87	534.20	281.99
7	−131.10	10.35	920.34	22.77	−22.28	373.81	382.05	86.79	−7.73	160.47
8	−131.07	10.36	920.37	22.69	25.39	155.84	269.92	494.51	250.83	91.03
9	−131.14	−314.64	760.42	−20.07	−21.66	208.30	108.93	−46.27	−149.05	140.26
10	−131.18	−314.62	760.31	−20.04	24.43	70.53	81.62	82.30	137.15	23.93
11	−131.18	−314.65	760.31	21.99	−21.60	230.52	242.87	−15.72	−224.92	−76.62
12	−131.15	−314.64	760.28	21.96	24.54	48.15	167.61	244.79	−18.86	−134.30
13	−1.14	−314.60	870.38	−22.40	−22.89	276.21	159.78	86.61	55.63	319.89
14	−1.12	−314.54	870.40	−22.27	26.99	62.52	119.61	243.10	398.26	142.93
15	−1.09	−314.62	870.40	23.73	−22.87	310.95	340.89	133.42	−53.23	−6.71
16	−1.11	−314.66	870.35	23.83	27.03	33.78	243.20	419.50	216.97	−88.72
17	−1.18	10.27	980.34	−17.67	−15.99	392.17	208.47	128.16	223.30	477.23
18	−1.19	10.27	980.37	−17.70	18.44	150.62	145.32	335.57	531.07	342.01
19	−1.12	10.12	980.40	16.57	−16.03	392.56	410.95	216.60	128.48	219.17
20	−1.09	10.11	980.37	16.48	18.43	150.40	306.45	498.95	351.80	142.71
21	−1.13	335.21	870.38	−22.17	−22.83	316.70	−0.99	−48.13	132.48	369.04
22	−1.16	335.28	870.29	−22.13	26.90	35.47	−74.39	215.56	435.53	250.94
23	−1.08	335.32	870.34	23.67	−22.77	276.35	259.43	61.16	89.33	154.93
24	−1.07	335.38	870.35	23.60	26.90	69.03	123.24	377.20	246.61	111.14
25	128.88	335.37	760.42	−20.27	−21.67	107.27	−145.35	−42.27	104.19	238.27
26	128.79	335.39	760.30	−20.23	24.63	−141.52	−200.57	136.70	313.90	135.60
27	128.82	335.34	760.29	21.99	−21.60	74.16	51.39	30.76	83.40	61.84
28	128.87	335.40	760.26	21.96	24.73	−113.15	−55.89	237.96	186.35	29.75
29	128.88	10.42	920.36	−21.20	−22.40	328.31	123.09	117.42	191.90	425.88
30	128.88	10.45	920.42	−21.06	25.50	−2.14	54.45	319.85	475.62	252.67
31	128.91	10.35	920.40	22.82	−22.36	324.25	372.08	196.54	114.75	118.27
32	128.93	10.32	920.35	22.93	25.54	0.68	237.55	461.95	320.03	41.48
33	128.82	−314.78	760.33	−19.87	−21.50	73.51	67.82	80.49	23.94	82.74
34	128.80	−314.74	760.38	−19.89	24.33	−119.69	36.82	184.44	244.00	−46.54
35	128.89	−314.87	760.41	22.17	−21.54	101.04	217.64	105.90	−47.03	−151.54
36	128.79	−315.10	760.23	22.00	24.27	−142.88	131.65	302.29	133.03	−211.36

Note: Angle unit: °, length unit: mm.

**Fig 13:**
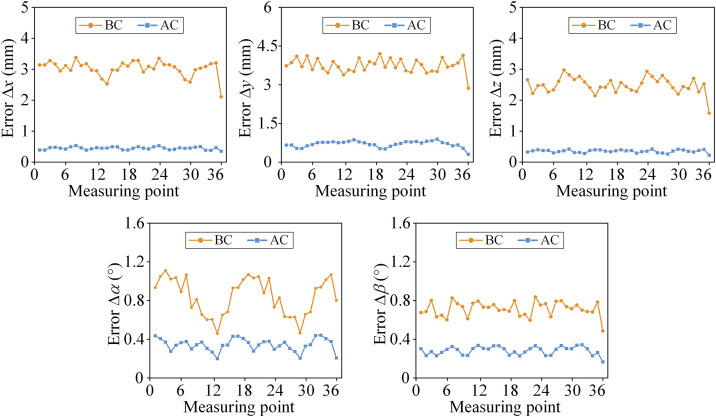
Errors of positions and postures before and after calibration compensation.

The error curves before calibration (BC) and after identification (AC) are obtained at the aforementioned measured points as shown in Fig 13.

It can be seen from Fig 13 that after the error compensation of manufacturing, assembly and actuator, the accuracy error is reduced from 3.41 mm, 4.18 mm, 2.97 mm, 1.11°and 0.84° to 0.53 mm, 0.88 mm, 0.42 mm, 0.43° and 0.34° in direction of the position *x*, *y*, *z*, angle *α* and *β*. The positioning accuracy of the five-degree-of-freedom direction of the parallel robot is improved by 84.4%, 78.9%, 85.8%, 61.2%, and 59.1%, respectively. The effectiveness of the calibration algorithm is verified through the experimental results.

A paired t-test [34] is employed to verify the statistical significance of the pose errors. Table 5 lists the p-values from the t-test for the errors in the five DOF before and after identification. The p-values from the paired t-test are all less than 0.05. It is concluded from Table 5 that considering the manufacturing and assembly errors of the components, the pose accuracy after kinematic calibration is acceptable.

**Table 5:** p-values of the paired t-tests in the five DOF directions.

Freedom direction	*t*	*p*
*x*	63.2492123769993	5.56698869303580e-38
*y*	60.3073254364646	2.90529360520355e-37
*z*	49.6607674371379	2.41149451739672e-34
*α*	18.4220716298725	6.82735670364796e-20
*β*	41.3926351741914	1.27385246363205e-31

**Fig 14:**
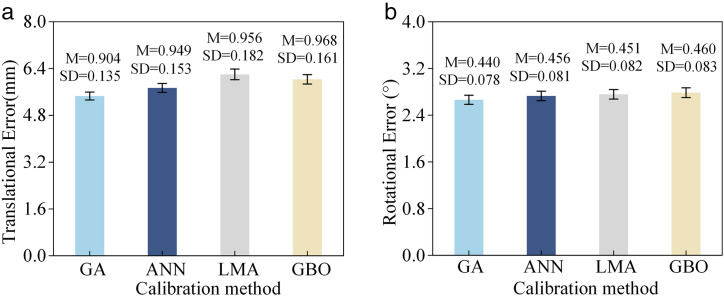
Pose errors of 5PUS-RPUR robot with different algorithms. **(a)** Position errors. **(B)** Posture errors.

To further validate the superiority of the proposed algorithm, a comparative study is conducted by performing kinematic calibration using artificial neural networks (ANN) [17], Levenberg-Marquardt algorithm (LMA) [16], and gradient-based optimizer (GBO) [35]. The identification parameters rather than the original parameters will be embedded into the control model. The mean and standard deviation of the errors are presented in Fig 14, where the scalar values for position and orientation errors are obtained via their Euclidean norms. It can be observed that the GA yields the lowest mean and standard deviation for both position and orientation errors, particularly in terms of position error. Although this superior accuracy is achieved at the expense of a higher computational cost, this is not a significant drawback, as offline calibration procedures are typically not subject to stringent time constraints.

The proposed self-calibration method is applicable to other parallel robots. The robot’s kinematic model is likewise established within the mathematical framework of screw theory, and an error model is constructed by means of the forward kinematics. By mounting sensors on the moving platform and the actuators, motion data from the actuated joints and the moving platform can be acquired. These data are then used in conjunction with a heuristic algorithm to identify the error parameters.

## 5. Conclusions

In order to improve the pose accuracy of the 5PUS-RPUR parallel robot, a method of kinematic error analysis and identification of the parallel robot is proposed. The main conclusions are as follows.

1) Combined with the screw theory, PK subproblems and elimination method, the inverse displacement of the series chain is analyzed to obtain the analytical solution of the inverse kinematics of the parallel robot.
2) The farthest point sampling algorithm is utilized to select measurement points uniformly throughout the robot’s workspace. This strategy ensures a comprehensive coverage of global errors, which is beneficial for improving the precision of the identification process.
3) By constructing an objective function that incorporates both the actuator displacement errors from each chain with the overall pose error of the moving platform, the coupling effects between chain parameters can be indirectly eliminated. This approach facilitates a globally optimal error identification result, thereby enhancing the pose accuracy of the parallel manipulator in all directions.

Future work will address errors that arise under dynamic loading, taking into account non-geometric factors such as the robot’s elastic deformation, and vibration. This will be achieved by integrating real-time error estimation methods with the offline geometric error compensation framework established in the present study.

## Supporting information

S1 Table This table presents the specific data for the pose error of the 5PUS-RPUR parallel robot, as depicted in Fig. 14. (XLSX)
